# Regulatory sharing between estrogen receptor α bound enhancers

**DOI:** 10.1093/nar/gkaa454

**Published:** 2020-06-01

**Authors:** Julia B Carleton, Matthew Ginley-Hidinger, Kristofer C Berrett, Ryan M Layer, Aaron R Quinlan, Jason Gertz

**Affiliations:** Huntsman Cancer Institute, University of Utah, Salt Lake City, UT, USA; Department of Oncological Sciences, University of Utah, Salt Lake City, UT, USA; Huntsman Cancer Institute, University of Utah, Salt Lake City, UT, USA; Department of Biomedical Engineering, University of Utah, Salt Lake City, UT, USA; Huntsman Cancer Institute, University of Utah, Salt Lake City, UT, USA; Department of Oncological Sciences, University of Utah, Salt Lake City, UT, USA; BioFrontiers Institute, University of Colorado, Boulder, CO, USA; Department of Computer Science, University of Colorado, Boulder, CO, USA; Departments of Human Genetics and Biomedical Informatics, University of Utah, Salt Lake City, UT, USA; Huntsman Cancer Institute, University of Utah, Salt Lake City, UT, USA; Department of Oncological Sciences, University of Utah, Salt Lake City, UT, USA

## Abstract

The human genome encodes an order of magnitude more gene expression enhancers than promoters, suggesting that most genes are regulated by the combined action of multiple enhancers. We have previously shown that neighboring estrogen-responsive enhancers exhibit complex synergistic contributions to the production of an estrogenic transcriptional response. Here we sought to determine the molecular underpinnings of this enhancer cooperativity. We generated genetic deletions of four estrogen receptor α (ER) bound enhancers that regulate two genes and found that enhancers containing full estrogen response element (ERE) motifs control ER binding at neighboring sites, while enhancers with pre-existing histone acetylation/accessibility confer a permissible chromatin environment to the neighboring enhancers. Genome engineering revealed that two enhancers with half EREs could not compensate for the lack of a full ERE site within the cluster. In contrast, two enhancers with full EREs produced a transcriptional response greater than the wild-type locus. By swapping genomic sequences, we found that the genomic location of a full ERE strongly influences enhancer activity. Our results lead to a model in which a full ERE is required for ER recruitment, but the presence of a pre-existing permissible chromatin environment can also be needed for estrogen-driven gene regulation to occur.

## INTRODUCTION

Regulation of gene expression is a fundamental task underlying biological processes such as development and disease progression. Promoter-distal gene regulatory enhancers play a central role in metazoan gene regulation and contain binding sites for transcription factors (TFs) that recruit cofactors and influence gene expression. Most genes in the human genome are likely regulated by multiple enhancers (1,2). For example, the ENCODE consortium found that an average of 3.9 distal elements are involved in long-range interactions with each transcription start site (3). While multiple enhancers often combine to regulate gene expression, the molecular details of how these enhancers work together remains poorly understood, partially due to a paucity of functional studies. Understanding how multiple enhancers molecularly communicate with each other and their target gene promoter represents a major open question in gene regulation.

The most commonly observed model for how enhancers combine to regulate gene expression has them acting in an independent or additive manner, allowing for elements to evolve independently, which can lead to divergence in tissue-specific expression patterns and gene expression levels. As an example, multiple independent enhancers regulate the β-globin locus in mice (4). Enhancers can also act in a synergistic or cooperative manner to influence gene expression (5). Leddin *et al.* found that in order for PU.1 to bind at one of its upstream enhancers in myeloid cells and auto-regulate expression, a second enhancer must be active. This second enhancer likely maintains accessible chromatin at the neighboring enhancer, enabling PU.1 to bind (6). Enhancers can also work together to maintain a favorable 3D chromatin architecture and promote transcription factor recruitment, as observed at the *Igk* locus in B cells (7,8). However, the molecular details behind enhancer interactions and features that dictate independence and cooperativity remain relatively unknown.

Estrogen signaling through estrogen receptor α (ER) is a relevant model system to study combinatorial gene regulation. ER binds the genome in an estrogen-dependent manner, with the majority of binding occurring distally from promoters (9). The majority of genes up-regulated upon estrogen treatment have multiple ER binding sites nearby (10), indicating that multiple sites might be required for coordinating the transcriptional response to estrogen. We previously developed a CRISPR interference based method, termed enhancer interference (11), to study enhancer relationships and identified two types of collaborative enhancer relationships: (i) hierarchical, where one predominant site contributes the majority of the estrogen response and another supportive site can contribute only when the predominant site is active, and (ii) synergy, where a pair of sites is completely necessary for the estrogen response and neither site can contribute in isolation (10). Paradoxically, when the same ER binding sites are targeted by CRISPRa fusions in the absence of estrogens, the enhancers work independently to regulate gene expression (12). Taken together these findings lead to a model in which enhancers are cooperating in *cis*, positively impacting one another when activated by ER, but communicate independently with the target gene promoter when directly bound by a synthetic transcriptional activator. To resolve this apparent contradiction and advance our understanding of enhancer synergy, it is important to determine how these enhancers are molecularly cooperating when cells are treated with estrogen.

In this study, we explore how neighboring ER-bound enhancers impact one another. By functionally dissecting these relationships at two estrogen-responsive genes, we discovered regulatory sharing between ER-bound enhancers. Deletion of ER-bound enhancers decreased ER binding, histone acetylation, and chromatin accessibility at neighboring enhancers. Through the use of genome engineering approaches, we also investigated the role of sequence and genomic location in determining the contributions of these enhancers to estrogen-induced gene expression. We find that the presence of a full estrogen response element (ERE), ER’s preferred DNA binding sequence, in at least one enhancer is required for these genes’ transcriptional responses to estrogen. The location of the ERE containing enhancer region is also important; placement of an ERE within a region harboring histone acetylation prior to estrogen induction greatly increases the transcriptional response. Overall, we discovered that these ER-bound enhancers are cooperating at a molecular level to combine ER recruitment and permissive chromatin and drive the estrogen transcriptional response.

## MATERIALS AND METHODS

### Cell culture

Ishikawa cell lines containing deletions of individual ER binding sites were generated as described previously (10). Ishikawa cells and T-47D cells were maintained in RPMI 1640 (Gibco) supplemented with 10% FBS (Gibco) and 1% pencillin/streptomycin (Gibco). Cells were placed in hormone-depleted RPMI (phenol red-free RPMI-1640 supplemented with 10% Charcoal/Dextran treated fetal bovine serum (HyClone) and 1% penicillin-streptomycin) for at least 6 days prior to E2 treatment.

### ChIP-seq

Approximately 20 million cells were plated in 15 cm dishes the day before harvest for each cell line of interest. For ER ChIP-seq, a 1 h 10 nM E2 induction was performed prior to harvest. For Ishikawa H3K27ac ChIP-seq, an 8 h 10 nM E2 or DMSO treatment was performed prior to harvest as this timepoint exhibited significant changes in our previous study (10). For T-47D H3K27ac ChIP-seq, cells were harvested in either full RPMI media or hormone-depleted RPMI with no E2 added. To crosslink chromatin, 37% formaldehyde (Sigma-Aldrich) was added directly to the media for a final concentration of 1% and allowed to incubate at room temperature for 10 min. Glycine was added at a final concentration of 125 mM to stop crosslinking, and cells were washed with cold 1× PBS. Plates were scraped in Farnham lysis buffer with 1× protease inhibitor (Thermo Fisher). Chromatin was sonicated using an Active Motif EpiShear Probe Sonicator with 6 cycles of 30 s, at 40% amplitude, with 30 s of rest. ChIP was performed as previously described (13) using anti-ER (Santa Cruz HC-20) and anti-H3K27ac (Active Motif 39133) antibodies. Libraries were sequenced on an Illumina HiSeq 2500 as single-end 50 basepair reads. Reads were aligned to hg19 using bowtie (14) with the following parameters: -m 1 -t –best -q -S -l 32 –e 80 -n 2. MACS2 (15) was used to call peaks with a *P*-value cutoff of 1e–10 and the mfold parameter bounded between 5 and 50. The input control used in peak-calling was derived from the parental Ishikawa line. The broad peak calling feature was used for H3K27ac ChIP-seq. Bedtools (16) was used to count reads in peaks. A window of 2 kb centered on the summit was used for counting H3K27ac ChIP-seq reads within peaks and a window of 500 bp was used for ER ChIP-seq read counting. ChIP-seq signals of an enhancer deletion was compared to clones that were wild-type for that locus but harbored enhancer deletions nearby the other gene. For example, MMP17-1 enhancer deletions were compared to CISH-1 and CISH-2 deletions as controls. Additional ChIP-seq data (Figure 2C-D) was from the ENCODE project (3).

### ATAC-seq

For each of the clones, ∼250 000 cells were lysed and nuclei were harvested for transposition as described previously (17) following a 1 h treatment with E2 or DMSO. We performed a 1 h E2 treatment for ATAC-seq based on previous studies looking at E2 induced chromatin accessibility changes (18,19). Tn5 transposase with Illumina adapters was assembled as previously described (20). Libraries were sequenced on a HiSeq 2500 with single-end 50 basepair reads. Reads were aligned to hg19 using bowtie (14) and the following parameters: -m 1 -t–best -q -S -l 32 -e 80 -n 2. SAM files were converted to BAM files for peak calling using samtools (21). MACS2 (15) was used to call peaks without an input using the following command: macs2 callpeak –nomodel –shift -100 –extsize 200 –B –SPMR. Peaks were overlapped with ER binding sites and reads were counted using bedtools (16). ATAC-seq signals of an enhancer deletion was compared to clones that were wildtype for that locus but harbored enhancer deletions nearby the other gene.

### Knock in blunt ligation

To generate PCR products for knock-ins of entire ERBS, we designed PCR primers (Supplementary Table S1) to specifically amplify the 125–225 bp region for each site that was deleted using Cas9 with previously designed guide RNAs (10). The PCR primers (IDT) included phosphothiorate bonds between the first three nucleotides to prevent degradation (22). PCR products were purified using Ampure XP (Beckman Coulter) and Sanger sequenced (Genewiz) to verify the product.

To generate knock-ins at a given ERBS, Ishikawa cells were plated at a density of ∼300 000 cells per well in six-well plates and transfected the following day with 1650 ng Cas9 plasmid (Addgene 62988, a gift from Feng Zhang), 550 ng of each guide RNA (Supplementary Table S2), and 200 ng PCR product using FuGENE HD (Promega). At 2 days post transfection, the media was changed and supplemented with 1 μg/mL puromycin to select for transfected cells. After 1–2 days of selection, the media was again changed to allow cells to recover for at least 1 day prior to limiting dilution cloning. When cells became confluent, they were subjected to limiting dilution cloning to isolate individual colonies containing specific mutations. When colonies were sufficiently large (about 2 weeks following plating), approximately 96 colonies were picked for each knock-in experiment. Colonies were allowed to grow in a 24-well plate until confluent, at which point they were moved to a 48-well plate, and most of the cells were harvested for genomic DNA isolation. Genomic DNA was isolated using the ZR-96 Quick-DNA extraction kit (Zymo Research) and subject to PCR using primers outside of the regions of interest that contained Illumina tails for high-throughput sequencing (Supplementary Table S1). PCR products were cleaned up using ZR-96 DNA Clean and Concentrate kits (Zymo Research) and a second PCR was performed to attach barcodes. PCR products were pooled by region of interest using 2–3 μl of each reaction and the resulting pools of products were purified using a 1× Ampure XP cleanup (Beckman Coulter). Purified libraries were pooled and submitted for paired-end 150 cycle sequencing on an Illumina MiSeq. Reads were aligned to a custom library containing the inserts of interest using bwa (23) with the following parameters: bwa mem –M –t 2.

### RNA isolation and qPCR

To harvest RNA, we used a direct on-plate lysis of cells with 300 μL of Buffer RLT Plus (Qiagen) supplemented with 1% beta-mercaptoethanol (Sigma-Aldrich). Lysates were purified using the ZR-96-well Quick-RNA kit (Zymo Research). RNA was quantified using RiboGreen (ThermoFisher Scientific) on a Wallac EnVision plate reader (PerkinElmer) or on a Qubit 2.0 (ThermoFisher Scientific). Gene expression was quantified using Power SYBR Green RNA-to-CT 1-Step Kit (ThermoFisher Scientific) and a CFX Connect light cycler (BioRad). Each reaction contained 50 ng of RNA as starting material. As per kit instructions, 40 cycles of PCR were performed following a 30-minute cDNA synthesis. Primers for *CTCF*, *CISH*, and *MMP17* can be found in Supplementary Table S1.

### Analysis of ERBS clusters and their activity

To identify distances between ERBS, we used bedtools closest (16) on previously generated ChIP-seq data for ER following a 1 h E2 treatment (24). ERBS clusters were generated using bedtools merge with a distance of 10 kb on a file containing all ERBS. Controls were performed by randomly selecting 8621 (the number of ERBS) previously identified DNase I hypersensitive sites or CTCF ChIP-seq sites (24) and then performing the same cluster analysis.

### Quantification and statistical analysis

To quantify differences in ChIP-seq and ATAC-seq signal resulting from ERBS manipulations, we used two-way ANOVA. To quantify differences in E2-induced gene expression following genetic manipulation, we used two-way ANOVA. To quantify differences in fold change in response to E2 for up-regulated genes with specific numbers of ERBS clusters within 100 kb of their TSSs, we used Wilcoxon signed-rank tests. *P*-values from these tests can be found in the text and in the figure legends.

## RESULTS

### Collaborative regulation by neighboring ER-bound enhancers may be a pervasive feature of the estrogen transcriptional response

**Figure 1.**
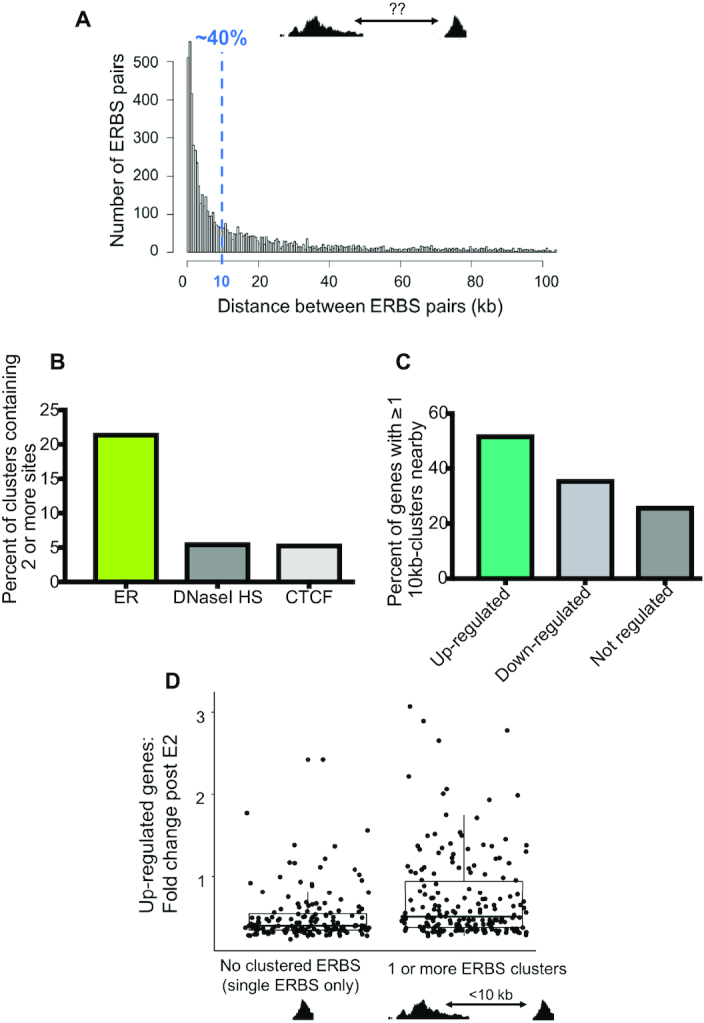
Clusters of ERBS are enriched near up-regulated genes. (**A**) Histogram depicts distance between an ERBS and its closest neighboring site for all 8621 sites bound in Ishikawa cells. (**B**) ERBS, CTCF sites bound in Ishikawa, and DNaseI hypersensitive sites (HS) were each merged into 10-kb windows throughout the genome and the number of windows containing multiple sites for each feature is shown. (**C**) The percent of up-regulated, down-regulated, and not regulated genes that have at least one cluster containing multiple ERBS is shown. (**D**) Boxplot shows the relationship between fold change in response to estrogen for up-regulated genes containing either dispersed sites (0 clusters) or at least 1 cluster containing multiple ERBS within 100 kb of the TSS.

*MMP17*, *FHL2* and *CISH* each harbor pairs of ER binding sites (ERBS) that work together synergistically (10). These ERBS pairs are within 5 kilobasepairs (kb) of each other, which led us to examine whether neighboring ERBS are a common feature of estrogen-regulated genes. To determine whether ER binding sites tend to cluster in the genome, we examined the distance between ERBS pairs using the set of 8621 ERBS bound in Ishikawa, a human endometrial cancer cell line, by ChIP-seq (24). We found that 42% of ERBS have a neighboring site within 10 kb (Figure 1A). In order to determine if this clustering of ERBS is a unique feature, we analyzed clustering of ERBS compared to controls of randomly subsampled DNaseI hypersensitive sites or CTCF binding sites in Ishikawa cells (8621 loci). We assigned each individual site to a window that contained 10 kb upstream and downstream of the site and sites within 10 kb of each other were merged into a cluster. We found that only 5–8% of windows for randomly sampled CTCF bound sites and DNaseI hypersensitive sites contained more than one site; however, 22% of windows had more than one ERBS (Figure 1B), indicating a substantial enrichment for clustering of ERBS compared to other regulatory regions. ERBS clustering has been previously observed in MCF-7 cells (25,26), a human breast cancer cell line, and our results indicate that ERBS cluster in endometrial cancer cells as well.

In order to better understand the relationship between clustered ERBS and gene expression, we examined where clusters are located in the genome relative to genes up-, down-, or not-regulated by estrogen as defined by RNA-seq (24). We found that 52% of up-regulated genes have a cluster of two or more ERBS nearby, compared to only 35% of down-regulated genes and 26% of not-regulated genes (Figure 1C). We then compared the fold change in 17β-estradiol (E2) response for up-regulated genes with no clusters to genes with at least 1 cluster within 100 kb of the transcription start site (TSS) and found that genes with clustered ERBS nearby tended to have greater fold changes in response to E2 (Figure 1D) (*P*-value = 3.93 × 10^−6^, Wilcoxon signed-rank test). These results suggest that ERBS clusters may be more effective at producing gene expression responses to estrogen compared to solitary ERBS.

### Neighboring ER-bound enhancers of CISH impact each other's ER occupancy and chromatin environment

**Figure 2.**
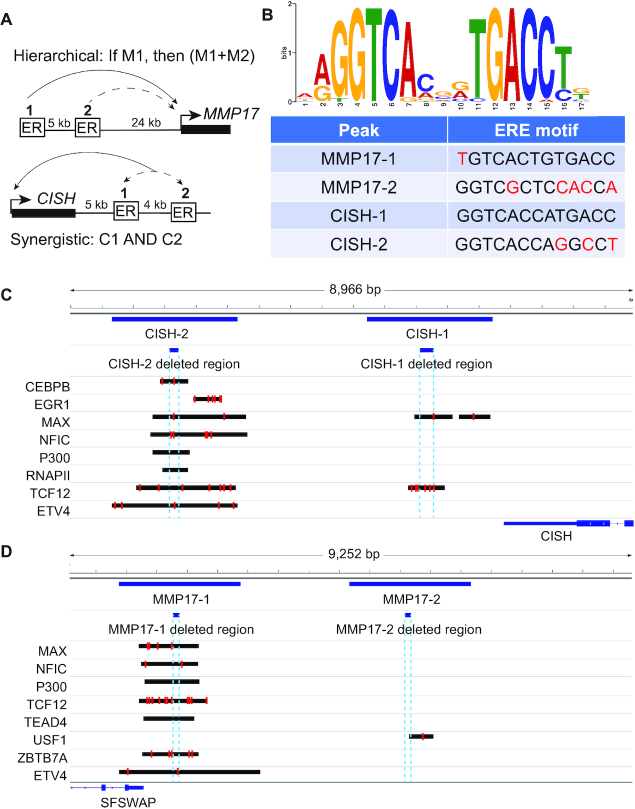
Features of enhancers controlling the estrogen transcriptional responses of *MMP17* and *CISH*. Hierarchical enhancers are upstream of *MMP17* (**A**). MMP17-1 contains a strong ERE (**B**, red letters indicate mismatches) and acts as the predominant site, while MMP17-2 has a half site and can contribute only when MMP17-1 is active. Synergistic enhancers are downstream of *CISH* (A). CISH-1 contains a canonical ERE motif (B) while CISH-2 does not. Both sites are equally necessary for the transcriptional response of *CISH*. (C, D) Browser tracks show binding of additional factors to *CISH* (**C**) and *MMP17* (**D**) ER-bound enhancers as well as the locations of the deleted regions (dashed blue lines) and locations of motif matches (red lines) found using FIMO (42) with a *P*-value cutoff of 0.001.

To determine how neighboring enhancers collaborate to produce an estrogen response, we focused on two ERBS near estrogen-responsive genes *CISH* and *MMP17* (Figure 2), which have previously been shown to be required for the transcriptional response to E2 in Ishikawa cells (10). Both genes contain a similar structure where one site in the pair has a full canonical estrogen response element (ERE) motif whereas the other site has a half ERE; however, the regulatory logic of each pair is a different type of non-independence (Figure 2A and B). At *CISH*, sites interact synergistically and both ERBS are equally necessary for the estrogen transcriptional response. At *MMP17*, a hierarchical relationship exists between ERBS, where one predominant site contributes the majority of the response and the other supportive site can contribute only when the predominant site is active (10). The ER-bound sites display differential binding of additional transcription factors (Figure 2C-D), which may in part shape their relationships to gene expression and to each other. To determine how each ERBS contributes to its respective pair, we generated two independent cell lines for each region of interest, with each line containing a deletion of one of the four ERBS. Deletions were generated using guide RNAs flanking the ER ChIP-seq peak, which removed 125–225 basepairs (bp) of sequence including potential binding motifs for ETV4 at MMP17-1 and CISH-2, CEBPβ, MAX, and NFIC at CISH-2, and TCF12 at CISH-1.

**Figure 3.**
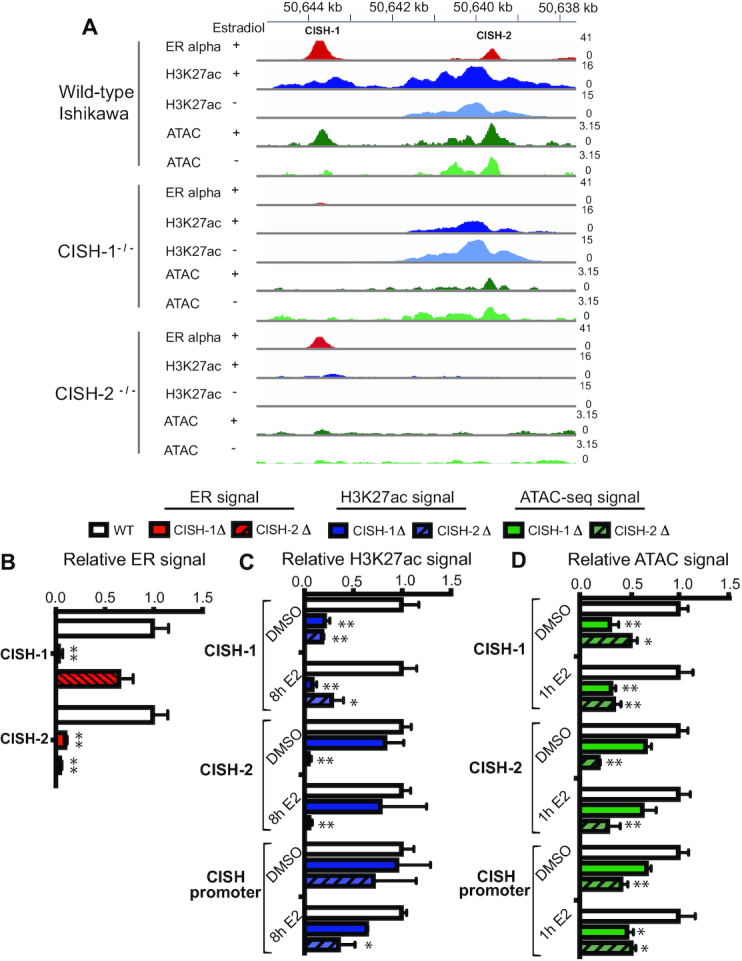
Synergistic enhancers divide tasks of recruiting ER and maintaining permissive chromatin. (**A**) Example of the effects of individual *CISH* enhancer deletions on ER binding (red) and H3K27ac (blue) at the neighboring enhancer as measured by ChIP-seq as well as ATAC-seq signal (green) in a representative clone. (**B**) ER ChIP-seq signal from 2 independent cell lines containing each deletion (solid red for CISH-1 and striped for CISH-2) was normalized to wild-type cells (white). (**C**) H3K27ac ChIP-seq signal from 2 independent cell lines containing each deletion (solid blue for CISH-1 and striped for CISH-2) and treated with either DMSO or 10 nM E2 for 8h was normalized to wild-type cells (white). (**D**) ATAC-seq signal from 2 independent cell lines containing each deletion (solid green for CISH-1 and striped for CISH-2) and treated with either DMSO or 10 nM E2 for 1h was normalized to wild-type cells (white). In B-D, error bars represent SEM, * indicates *P* < 0.05, and ** *P* < 0.001 in a two-way ANOVA. ATAC-seq and ChIP-seq signal at the *MMP17* promoter and control regions are shown in Supplemental Figure S1.

Since previous work has found that an enhancer can affect chromatin and TF binding at a neighboring enhancer, we hypothesized that ER-bound enhancers may affect each other via similar mechanisms. We performed ATAC-seq and ChIP-seq for ER and H3K27ac in the enhancer deletion lines after treatment with E2 or vehicle (DMSO). As expected, deletion of a central 225 bp of CISH-1 led to a complete loss of ER binding and large reductions in accessibility and H3K27ac at CISH-1 compared to wild-type levels (Figure 3A–D). Loss of CISH-1 also led to a 90% reduction in ER binding at CISH-2 (Figure 3B); however, H3K27ac at CISH-2 was not significantly affected either with or without E2 treatment (Figure 3C). Similar to H3K27ac, CISH-1 deletion reduced accessibility of CISH-2 by approximately 33% (*P*-value > 0.05) regardless of treatment (Figure 3D). These results suggest that CISH-1 participates in the synergistic relationship between CISH-1 and CISH-2 by controlling ER binding to the locus, but minimally contributes to accessible and permissive chromatin of the enhancer neighborhood.

We next investigated how enhancer CISH-2 contributes to the synergistic relationship at *CISH*. Deleting 166 bp of CISH-2 resulted in loss of ER binding, H3K27ac and chromatin accessibility at CISH-2 as expected (Figure 3A–D). Loss of CISH-2 caused ER signal at CISH-1 to be reduced by 34% (*P*-value > 0.05) with ER binding still detectable (Figure 3B). Deletion of CISH-2 led to loss of H3K27ac signal at CISH-1, with or without E2 induction, to levels seen upon CISH-1 loss, indicating that CISH-2 is necessary for permissive chromatin at CISH-1 (Figure 3C). In addition, CISH-2 loss had a greater effect on *CISH* promoter H3K27ac levels than CISH-1 loss with or without E2 treatment (Figure 3C), suggesting that CISH-2 may be responsible for H3K27ac levels at the entire locus. Accessibility of CISH-1 was also significantly affected by CISH-2 loss, with a 50% loss in the absence of estrogens and a 66% loss in the presence of estrogen (Figure 3D). CISH-2 deletion had a greater impact on promoter accessibility prior to E2 treatment; however, CISH-1 and CISH-2 were equally necessary for promoter accessibility in the presence of estrogen, with a 50% reduction in accessibility in the absence of either site (Figure 3D). CISH-2 exhibits open and acetylated chromatin prior to E2 treatment, while CISH-1 does not, indicating that these features may be important for CISH-2 function. These results suggest that the synergy between CISH-1 and CISH-2 is mediated by the sharing of ER recruitment and the maintenance of active and accessible chromatin between enhancers. CISH-1 contributes ER recruitment, which is consistent with the presence of a full ERE, while CISH-2 promotes a permissive chromatin environment. Altogether, the results at *CISH* indicate that recruiting ER to the genome is not sufficient for an ERBS to produce the estrogen response: some level of histone acetylation and accessibility, here provided by CISH-2, is required for a site to contribute to gene regulation.

### ER binding and permissive chromatin at supportive enhancer MMP17-2 require predominant enhancer MMP17-1

**Figure 4.**
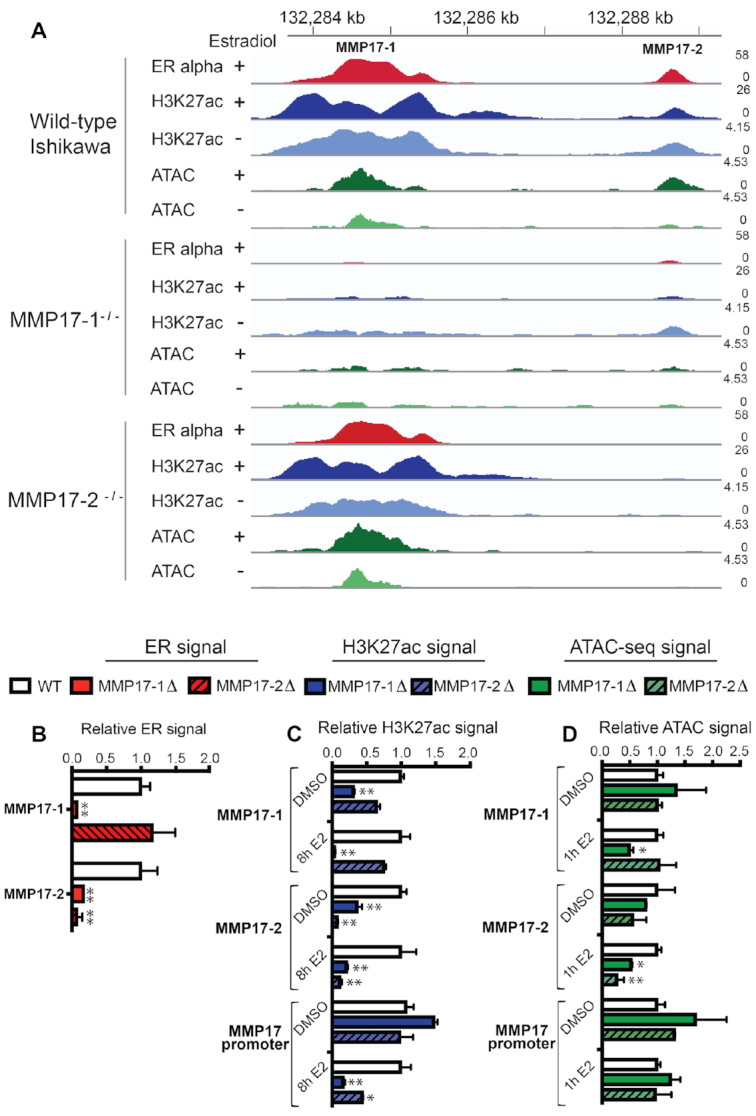
Predominant ERBS regulating *MMP17* controls ER binding and activity at the neighboring site. (**A**) Example of the effects of individual *MMP17* enhancer deletions on ER binding (red) and H3K27ac (blue) at the neighboring enhancer as measured by ChIP-seq as well as ATAC-seq signal (green) in a representative clone. (**B**) ER ChIP-seq signal from 2 independent cell lines containing each deletion (solid red for MMP17-1 and striped for MMP17-2) was normalized to wild-type cells (white). (**C**) H3K27ac ChIP-seq signal from 2 independent cell lines containing each deletion (solid blue for MMP17-1 and striped for MMP17-2) and treated with either DMSO or 10 nM E2 for 8h was normalized to wild-type cells (white). (**D**) ATAC-seq signal from two independent cell lines containing each deletion (solid green for MMP17-1 and striped for MMP17-2) and treated with either DMSO or 10 nM E2 for 1h was normalized to wild-type cells (white). In B–D, error bars represent SEM, * indicates *P* < 0.05, and ** *P* < 0.001 in a two-way ANOVA. ATAC-seq and ChIP-seq signal at the *CISH* promoter and control regions are shown in Supplemental Figure S1.

We next explored the molecular underpinnings of the hierarchical relationship between enhancers at *MMP17*. Loss of 150 bp of MMP17-1 abolished ER binding and greatly reduced H3K27ac and ATAC-seq signal after estrogen treatment at MMP17-1 as expected (Figure 4A–D). MMP17-1 deletion reduced ER binding at MMP17-2 by 79% compared to wild-type levels (Figure 4A and B). MMP17-1 deletion also greatly reduced H3K27ac levels at MMP17-2, leading to a 64% reduction in the absence of E2 and a 78% reduction in the presence of E2 (Figure 4C). These results suggest that MMP17-1 controls both permissive chromatin and ER recruitment at MMP17-2. Chromatin accessibility in the absence of E2 was not significantly affected by MMP17-1 loss, but the accessibility of both MMP17-1 and MMP17-2 was reduced by half after E2 treatment (Figure 4D). At the promoter of *MMP17*, histone acetylation levels were reduced by 85% upon MMP17-1 loss in the context of E2 treatment, while promoter accessibility was not significantly affected in either condition, suggesting that other factors are likely involved in maintaining open chromatin at the promoter. Together these results demonstrate that MMP17-1 is at the top of the hierarchy because it orchestrates ER recruitment (through a full ERE), histone acetylation, and, to a lesser extent, chromatin accessibility of MMP17-2, explaining why MMP17-1 is needed in order for MMP17-2 to contribute to the estrogen response.

We next examined how MMP17-2 might be acting as a supportive site at the locus. We found that loss of 128 bp of MMP17-2 did not significantly affect ER binding at MMP17-1, indicating that it is not required for the initial recruitment of ER to the locus (Figure 4A and B). MMP17-2 deletion resulted in a 35% reduction of H3K27ac at MMP17-1 in the absence of estrogens (Figure 4C), suggesting that MMP17-2 loss affects MMP17-1 to some extent. However, ER binding at MMP17-1 appears to be able to partially compensate for the reduced histone acetylation, as MMP17-2 loss does not significantly reduce H3K27ac levels at MMP17-1 after E2 treatment. Deletion of MMP17-2 led to 55% reduction of H3K27ac at the *MMP17* promoter in the presence of E2, indicating that loss of MMP17-2 does affect the promoter; however, chromatin accessibility is not significantly impacted by MMP17-2 loss, similar to MMP17-1 loss (Figure 4D). This data leads to a model in which MMP17-1 is responsible for ER recruitment and permissive chromatin in the neighborhood. MMP17-1 is able to confer these features to MMP17-2, allowing MMP17-2 to contribute to gene regulation as well.

### DNA sequence is critical in determining how an ER-bound enhancer contributes to gene expression

The analysis of enhancer deletions uncovered a molecular interplay between enhancers with ERBS contributing ER recruitment and/or active and accessible chromatin to their neighborhood. We next sought to determine the ERBS features that govern each site's contribution to gene expression, considering two possibilities: 1) The underlying DNA sequence is dictating the contribution of a site, or 2) the genomic location of a site determines the site's role in gene regulation. To investigate the importance of these features, we used Cas9 to edit the sequence of ERBS at *MMP17* and *CISH* to match the core ∼200 bp of the neighboring enhancer. We used Knock-in Blunt Ligation (22), which relies on double-stranded break repair by non-homologous end joining to insert a PCR product into the desired locus. While the orientation of the insert cannot be controlled this way, full EREs are palindromic sequences that lack orientation and enhancers can work in either orientation, suggesting that orientation should not have a large impact on gene regulation.

**Figure 5.**
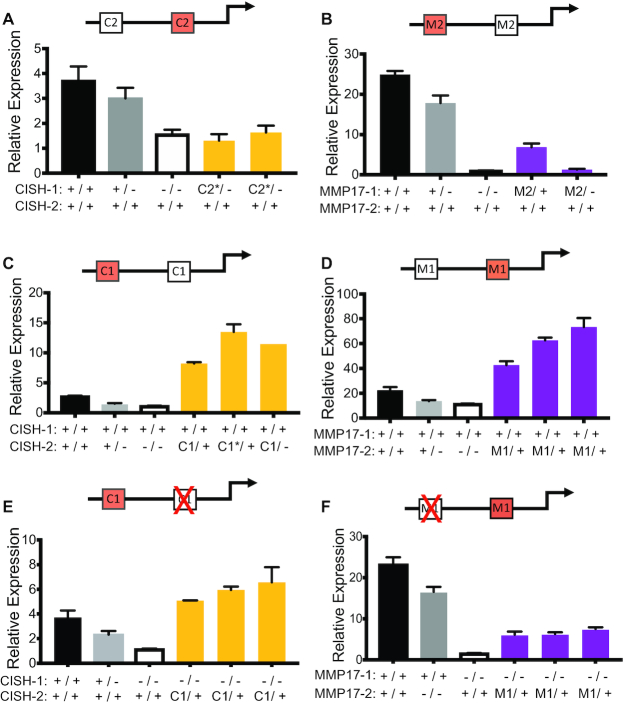
ERBS sequence and location are both important in driving the transcriptional response to estrogen. Red sites on the diagrams have been knocked-in and a red ‘X’ represents deletion. (**A–D**) The impact on the transcriptional response to E2 of replacing one allele of an enhancer with the sequence of the neighboring enhancer. This includes replacing CISH-1 with the sequence of CISH-2 (A, yellow bars), MMP17-1 with the sequence of MMP17-2 (B, purple bars), CISH-2 with the sequence of CISH-1 (C, yellow bars), and MMP17-2 with the sequence of MMP17-1 (D, purple bars). Wild-type (black), heterozygous deletion (gray), and homozygous deletion (white) clones are shown for each gene. (**E**) The gene regulatory impact of replacing one allele of CISH-2 with the sequence of CISH-1 (yellow bars) in the absence of wild-type CISH-1 is shown for multiple independent clones as well as wild-type (black), heterozygous CISH-1 deletion (gray), and homozygous CISH-1 deletion clones (white). (**F**) The gene regulatory impact of replacing the sequence of MMP17-2 with MMP17-1 when wild-type MMP17-1 is deleted is shown for multiple independent clones as well as wild-type (black), MMP17-2 deletion (gray), and MMP17-1 deletion (white) clones. In all panels, bars represent qPCR data from two biological replicates following an 8h estrogen induction and expression is relative to an 8h vehicle treatment for each clone. * indicates opposite orientation of insertion in terms of native locus relative to the target gene.

We created two independent cell lines where one allele of CISH-1 had been replaced with the core sequence of CISH-2. For both of these cell lines, the other allele of CISH-1 was deleted. We compared the estrogen response of these lines to wild-type lines as well as clones containing deletions of CISH-1. We found that the insertion of CISH-2’s sequence did not create an estrogen response in the absence of CISH-1’s core sequence in either clone (Figure 5A), indicating that the sequence of CISH-1 is required for an estrogen response. However, it is important to point out that each clone analyzed had a reverse sequence orientation and since CISH-2 harbors a non-palindromic half ERE, the reverse orientation could impact ER binding or regulatory activity. Similarly, at *MMP17*, replacing one MMP17-1 allele with the core sequence of MMP17-2 led to a loss of the estrogen response when the other MMP17-1 allele was deleted (Figure 5B). Even when one MMP17-1 allele was present with MMP17-2 on the other allele, the response was reduced by 75% compared to wild-type levels, indicating that a fully wild-type MMP17-1 sequence is needed for the estrogen transcriptional response. These results are consistent with our analysis of enhancer deletions, where the full EREs present in MMP17-1 and CISH-1 are required for ER recruitment to the neighborhood and half EREs cannot compensate for the lack of a full ERE.

We next examined the effects of replacing CISH-2, which maintains active and accessible chromatin in the neighborhood, with the core sequence of CISH-1. When one allele of CISH-2 was replaced with the core sequence of CISH-1, the estrogen response more than doubled relative to wild-type levels in all three clones we isolated (Figure 5C). Similarly, replacing MMP17-2 with the core sequence of MMP17-1 resulted in a doubling or more in expression levels of *MMP17* compared to the wild-type response for all three clones (Figure 5D). These results suggest that the wild-type expression of *MMP17* and *CISH* is suboptimal in terms of levels, as greater expression levels can be achieved when a sequence with a second full ERE is added to a neighborhood. These results also highlight the importance of local DNA sequence in determining ERBS function.

### Genomic location influences the activity of ER-bound enhancers

While DNA sequence is clearly important in determining an ERBS’s contribution to gene regulation, the genomic location of that sequence could also play a large role in the ability of an ERBS to regulate gene expression. To uncover the importance of location in ERBS activity, we next deleted the neighboring wild-type site in the DNA sequence swapped cell lines described above. This approach created alleles in which the core sequence of an ER-bound enhancer had been moved to the neighboring ERBS location without the influence of another ERBS. These manipulations allowed us to specifically examine the effect of a new location for a given ERBS sequence. While CISH-1 in its native location is unable to support an estrogen response when CISH-2 is deleted, moving the core sequence of CISH-1 into the CISH-2 location was sufficient to drive an estrogen response that is greater than wild-type levels, even without another ERBS in the neighborhood (Figure 5E). The ability of a single copy of CISH-1’s core sequence to drive higher expression in CISH-2’s location than in its native location indicates that while CISH-1 may be an optimal sequence for inducing an E2 transcriptional response, its location is suboptimal, potentially due to the lack of H3K27ac at CISH-1 prior to an estrogen treatment.

When we previously deleted MMP17-2, we found that the predominant site MMP17-1 can drive approximately half of *MMP17*’s response when acting alone. However, when MMP17-1’s core sequence is moved from its original location to MMP17-2’s location, this sequence was unable to drive expression at levels achieved by wild-type MMP17-1 without the support of MMP17-2 (Figure 5F). While the clones with the MMP17-1 location change could still contribute to the E2 response to some extent, the level is significantly less than half of MMP17-2 homozygous deletion clones (*P*-value = 0.0004, *t-*test). These results indicate that MMP17-1 is an optimal location for the underlying optimal sequence of MMP17-1 and that MMP17-2’s location is suboptimal. Interestingly, both optimal locations (MMP17-1 and CISH-2) are in open chromatin surrounded by high levels of H3K27ac prior to estrogen induction, suggesting that a combination of permissive chromatin and DNA sequence (with a full ERE) combine to drive an estrogen response.

Since H3K27ac is observed at MMP17-1 and CISH-2 when cells are starved for estrogens, it is likely that other transcription factors are responsible for maintaining histone acetylation at certain ERBS. To identify which factors might contribute to permissive chromatin at ERBS across the genome, we performed differential motif enrichment analysis using AME from the Meme Suite (27). In ERBS that do not overlap regions of H3K27ac, we found a strong enrichment for an ERE, which is expected based on the previous finding that ERBS with strong EREs are often in closed chromatin (24), as well as a homeodomain binding motif (Supplementary Figure S2A). In ERBS that overlap H3K27ac, we found several enriched motifs including a GC-rich sequence that matches SP1, KLF5, and EGR1 binding motifs, an NFI motif, an e-box, and an ETS motif (Supplementary Figure S2B). Analysis of ENCODE (3) ChIP-seq data confirmed a significant enrichment of transcription factors that bind to motifs enriched at ERBS with H3K27ac compared to ERBS without H3K27ac, including EGR1, NFIC, MAX, TCF12, USF1, ETV4 and SRF. These factors, with the exception of SRF and USF1, bind MMP17-1 and/or CISH-2 (Figure 2). The identification of transcription factors bound to ERBS in regions of histone acetylation indicate that additional transcription factors may aid in the transcriptional response to estrogen. Another indication that the estrogen transcriptional response is controlled in part by other factors is the cell type-specific regulation of *CISH* and *MMP17*. We performed ChIP-seq for H3K27ac in T-47D cells, an estrogen responsive breast cancer cell line. We also analyzed T-47D expression levels of *CISH* and *MMP17* using RNA-seq (24). The gene expression response and histone acetylation pattern near *CISH* are very similar between Ishikawa and T-47D (T-47D fold change = 2.56; Ishikawa fold change = 2.89) (Supplementary Figure S3). However, *MMP17* expression is not induced by E2 treatment in T-47D (T-47D fold change = 1.12; Ishikawa fold change = 14.23) and the MMP17-1 and MMP17-2 enhancers are devoid of H3K27ac and ER binding in T-47D cells. Together, these results highlight a potential contribution of other transcription factors to an estrogen transcriptional response.

### MMP17-like active clusters are common across the genome

**Figure 6.**
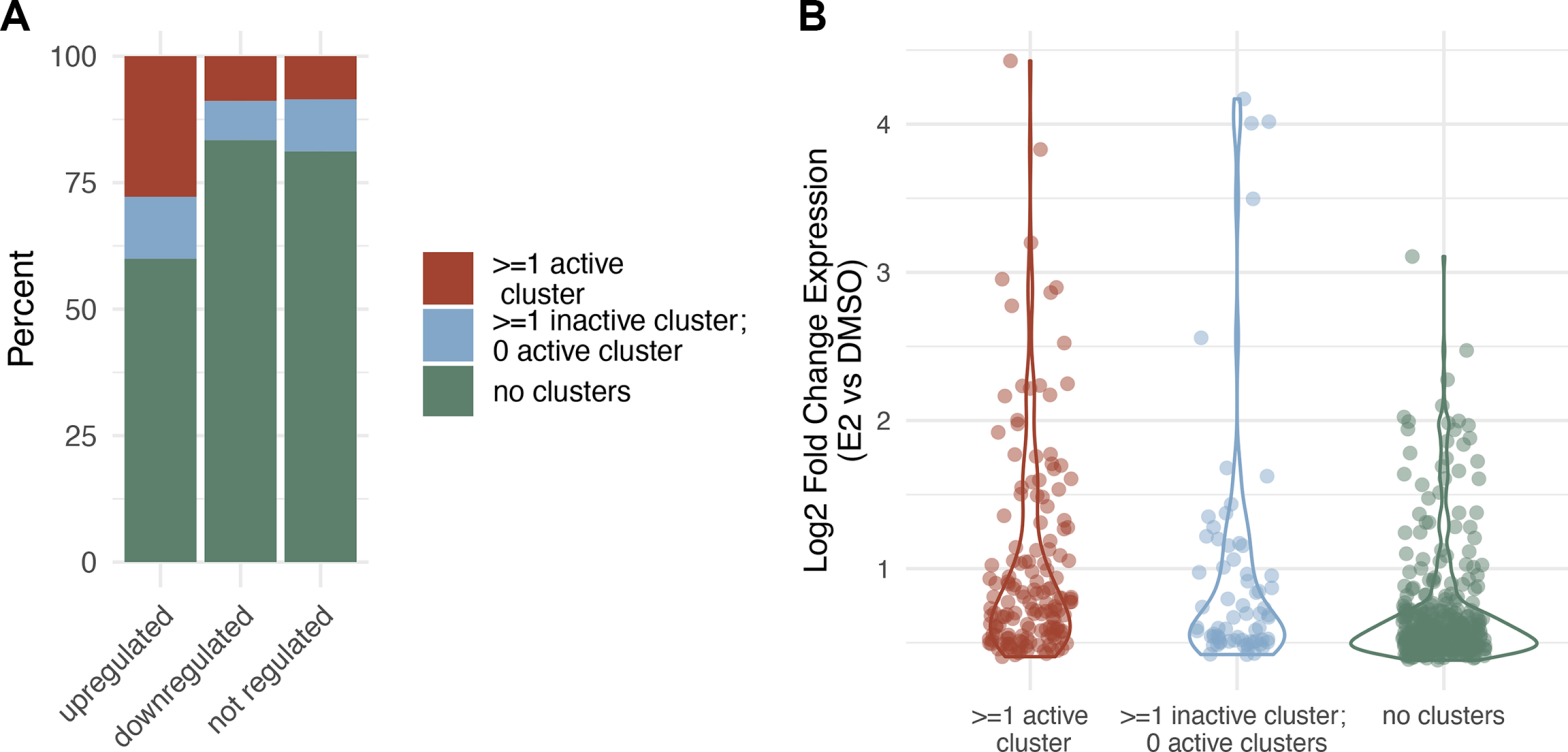
Active clusters are found near up-regulated genes with larger expression changes. (**A**) Bar graph shows the percent of up-regulated, down-regulated, and not regulated genes that harbor at least one active ERBS cluster (red), at least one inactive ERBS cluster and no active ERBS clusters (blue), and no ERBS clusters (green) within 100 kb of their transcription start sites. (**B**) Log_2_ expression fold changes in response to an 8-hour E2 treatment are shown for up-regulated genes partitioned based on ERBS clusters within 100 kb of their transcription start sites.

Based on the regulatory sharing observations at *CISH* and *MMP17*, we revisited the genome-wide analysis of ERBS clusters. Our functional dissection of ERBS clusters near *MMP17* and *CISH* indicated that two features – a strong ERE motif and histone acetylation prior to estrogen treatment—are important for a cluster to robustly contribute to gene expression. We explored whether other clusters had similar features, and whether these features were associated with the transcriptional response to estrogen. We examined all 6211 clusters generated above, including those containing only one ERBS, and determined whether the sites within that cluster had the combination of at least one full ERE and at least one H3K27ac site in the absence of E2, which we refer to as active clusters. To investigate how these active ERBS clusters relate to gene expression, we examined the localization of active clusters relative to genes up-, down-, and not-regulated by estrogen. We found that 28% of up-regulated genes have at least one active cluster within 100 kb, compared to 9% for down-regulated genes and 9% for not-regulated genes (*P*-value = 1.7 × 10^−14^, up vs. down; *P*-value < 2.2 × 10^−16^, up versus not; Fisher's exact test) (Figure 6A). Active clusters are more enriched nearby up-regulated genes than inactive clusters, with 28% of genes near active clusters and 12% of genes near inactive clusters (*P*-value = 1.5 × 10^−11^, odds ratio = 2.7, Fisher's exact test). When we compared transcriptional responses to E2 for up-regulated genes with active clusters nearby to up-regulated genes without active clusters nearby (Figure 6B), we found that significantly larger fold changes are observed for genes with active clusters nearby (*P*-value = 1 × 10^−8^, Wilcoxon signed-rank test). These results suggest that active clusters are more likely to drive larger estrogen transcriptional responses than inactive clusters, but that many genes are up-regulated by E2 without an ERBS cluster within 100 kb that contains both a full ERE and H3K27ac prior to E2 treatment.

We next sought to determine whether these active clusters near up-regulated genes looked more similar to the synergistic enhancers of *CISH* or the hierarchical enhancers of *MMP17*. To identify potential hierarchical sites (MMP17-like), we looked for clusters that had an ERBS that harbored both a strong ERE and H3K27ac in the absence of estrogens. To identify potential synergistic sites (CISH-like), we looked for clusters where one ERBS had a strong ERE and another ERBS had H3K27ac in the absence of estrogens. The vast majority of active clusters exhibited the hierarchical patterns seen at *MMP17* (82%). When focused solely on ERBS clusters near up-regulated genes, 113 genes have an MMP17-like ERBS cluster, 23 genes have a CISH-like ERBS cluster, and 16 genes have both types of clusters within 100 kb. These findings suggest that a hierarchical relationship between ERBS is the more common type of collaboration between ER-bound enhancers based on associated features.

## DISCUSSION

The ER-bound enhancers that we have previously studied in detail are within 5 kb of one another and combine cooperatively to regulate gene expression in response to estrogen. In this study we find that ER-bound sites in endometrial cancer cells tend to cluster together in the genome much more than expected by chance, as previously reported in breast cancer cells (25,26), suggesting that enhancer collaboration between neighboring ERBS could be common. In order to determine how ER-bound enhancers are impacting one another, we analyzed cell lines with homozygous deletions of two pairs of neighboring enhancers that regulate *CISH* and *MMP17*. We discovered molecular interplay between neighboring enhancers, which provides an explanation for the collaboration that we see between ER-bound enhancers.

One way in which these enhancers impact their neighbors is through recruitment of ER, despite kilobasepairs of sequence in between them. Such cooperativity is common when binding motifs are immediately adjacent to one another. For example, deletion of a single binding site for OCT4/SOX2 within an enhancer near the murine *Klf4* locus prevents other transcription factors from binding to that same enhancer and reduces chromatin accessibility at the enhancer (28). Enhancers can also impact each other at larger distances, such as *PU.1* autoregulation in myeloid cells, where C/EBPa binds to an enhancer 14 kb upstream of *PU.1* and promotes the activation and accessibility of a neighboring enhancer 2 kb downstream of the predominant enhancer (6). Similar enhancer hierarchies between individual enhancers have recently been described in other systems (29,30), and our genome-wide observations suggest that these hierarchies are likely a consistent feature of estrogen-dependent gene regulation.

For both *MMP17* and *CISH*, the enhancer required for bringing ER to the ERBS cluster contained a full ERE, while the ERBS with a half ERE was dispensable for ER binding. When the DNA sequences of full ERE sites were replaced with half ERE containing sequences, the gene expression response to estrogen was lost. Alternatively, swapping a half ERE containing core sequence with a full ERE containing sequence resulted in a super-response to estrogen for both genes. While this indicates that the enhancer sequences of *MMP17* and *CISH* are suboptimal in terms of levels, the combination of sequences allows for fine tuning of an estrogen response through evolutionary changes in both sequence and ERE location. The importance of a full ERE in a cluster is consistent with findings in mice expressing ER DNA binding domain mutants, where only consensus EREs with 1–2 mismatches contribute to gene expression (31). In addition, an analysis of ER-bound ‘super enhancers’ found that ERE motif strength is higher in regions that precede ‘super enhancer’ formation (25). Our findings indicate that full EREs are necessary for ER recruitment and gene expression, although we also found a role for sites with half EREs. Since our intensive study was limited to two genes, it is possible that multiple half ERE ERBS can combine to regulate expression. Previous studies have found that multiple suboptimal TF binding motifs can drive expression of reporters in *Ciona* and *Drosophila* (32,33). However, multiple weak TF binding motifs have yet to be manipulated in the genome simultaneously, and their ability to contribute in enhancer pairs remains unclear.

In addition to neighboring ERBS contributing to each other's ability to bind ER, ER-bound enhancers can also impact one another's chromatin environment. CISH-2 and MMP17-1 were needed to maintain histone acetylation and chromatin accessibility in their respective neighborhoods. Clustered ERBS can aid each other in maintaining permissive chromatin and this chromatin environment appears crucial. When core sequences of full ERE enhancers were moved to different locations, we found that placing a full ERE sequence in a region with high basal histone acetylation had a larger impact on transcription than adjacent regions with lower histone acetylation. Our results suggest that sites contributing to the transcriptional response to estrogen need some level of permissive chromatin prior to an estrogen induction, whether it is at the same site as the full ERE (e.g. MMP17-1) or the neighboring site (e.g. CISH-2). These findings are consistent with previous work showing that recruitment of p300 and subsequent histone acetylation appears to be a crucial step in estrogen-dependent gene regulation, as inhibitors of the p300 catalytic domain abrogate estrogen-dependent transcription (34). The fact that permissive chromatin in the absence of estrogens appears important suggests that these sites may be primed for activation; however, it remains unclear what factors are involved in priming these sites. Analysis of additional factors bound to these regions indicates that p300 is bound to CISH-2 and MMP17-1, the sites responsible for acetylation of the neighborhood. NFIC and ETV4 (35) were also uniquely found at CISH-2 and MMP17-1 as opposed to CISH-1 and MMP17-2. An expanded genome-wide analysis of ERBS, split by H3K27ac overlap, uncovered strong enrichment of EGR1, NFIC, MAX, TCF12, USF1, ETV4 and SRF at ERBS that overlap with H3K27ac compared to ERBS without H3K27ac. Many of these transcription factors have been shown to recruit histone acetyltransferases (36–41), consistent with their potential role in maintaining histone acetylation at certain ERBS. In addition, the cell type specificity in regulation of *MMP17*, but not *CISH*, reinforces a role for chromatin environment and the actions of other transcription factors in shaping the transcriptional response to estrogen.

**Figure 7.**
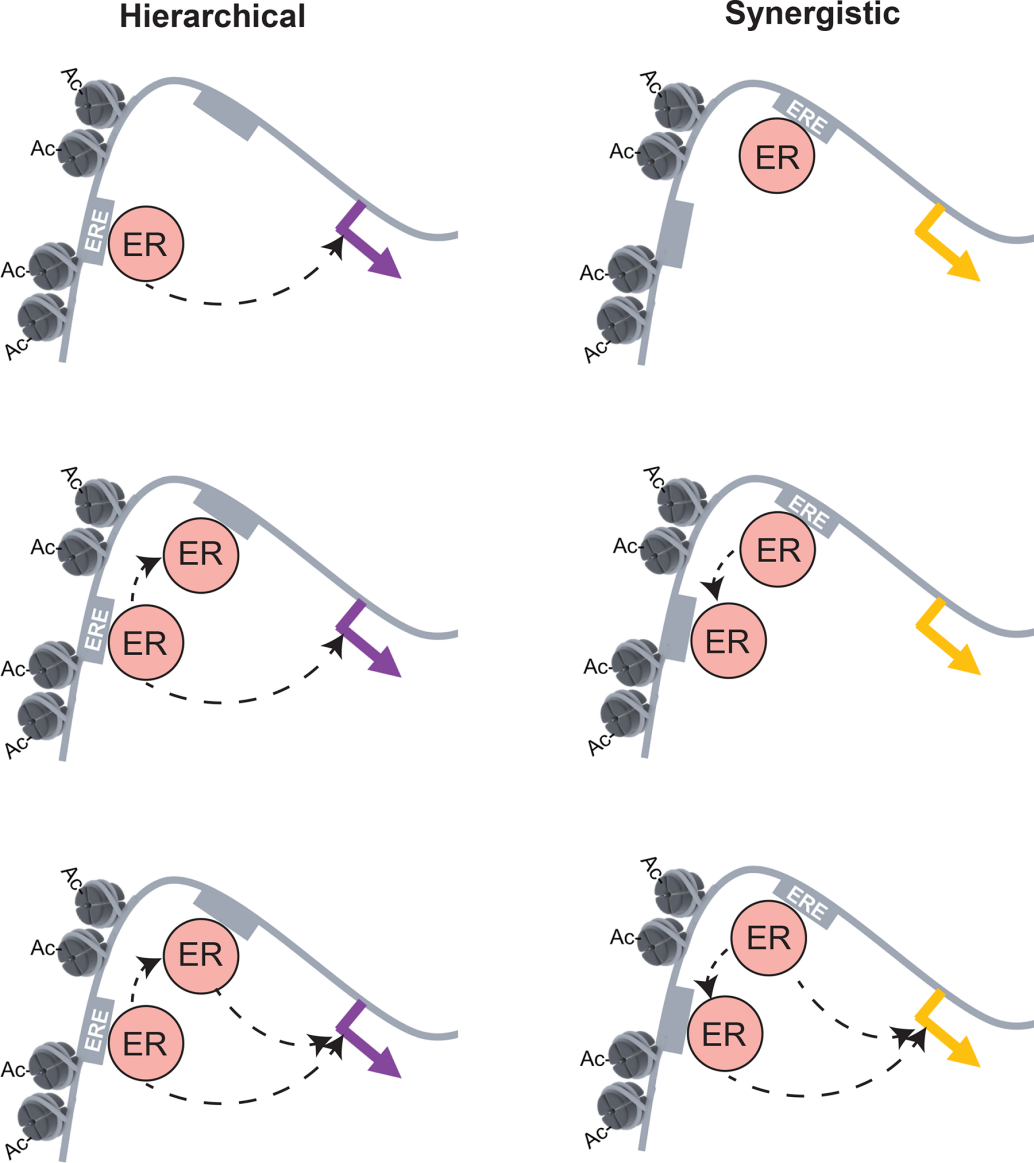
Model for two distinct modes of enhancer collaboration at neighboring ERBS. In a hierarchical relationship, a site in permissive chromatin and containing a consensus ERE motif can directly recruit ER and regulate gene expression. Only after this site becomes active can ER bind at the neighboring site that contains a weak ERE. This site contributes to gene expression by affecting activity of the promoter. In a synergistic interaction, the strong ERE site can recruit ER, but cannot contribute to expression without the presence of the neighboring weak ERE site. The presence of the weak ERE site promotes permissive chromatin in the region while also acting as a second binding site for ER, allowing both sites to contribute to gene expression.

When taken together, our observations provide an explanation for the synergistic relationship of ERBS regulating *CISH* and the hierarchical relationship of enhancers regulating *MMP17* (Figure 7). MMP17-1 is responsible for both ER recruitment and a permissible chromatin environment, which is consistent with its role atop the hierarchy. The activity of MMP17-1 allows MMP17-2 to bind ER and adopt a permissible chromatin context. CISH-1 and CISH-2 exhibit balanced regulatory sharing, which explains the complete synergy observed in their activation of *CISH* expression. CISH-1 contributes ER recruitment while CISH-2 provides permissive chromatin and both molecular actions are required for the estrogenic transcriptional response of *CISH*. The molecular mechanisms of how both sets of enhancers work together in *cis* reconciles previously disparate observations concerning these ER-bound enhancers: When ER is driving the transcriptional response, enhancers work cooperatively (10) and they do so by contributing to each other's activity; however, when activating cofactors are directly recruited to these sites, synergy is lost (12) because the cooperative steps (ER recruitment, chromatin accessibility, and histone acetylation) have been bypassed. Genome-wide analysis that took sequence and chromatin patterns into account, found that active ERBS clusters are more likely to be found nearby up-regulated genes and are associated with larger expression changes. This analysis also revealed that a large majority of active ERBS clusters are MMP17-like, suggesting a hierarchical relationship, and very few clusters have CISH-like features of synergy. While the MMP17- and CISH-like patterns are seen throughout the genome, most genes do not have an active cluster within 100 kb that resembles either MMP17 or CISH enhancers, which suggests that other modes of regulation through ER-bound enhancers likely exist that were not observed during the in-depth analysis of *CISH* and *MMP17* enhancers.

## DATA AVAILABILITY

Raw and processed sequencing data can be found at the Gene Expression Omnibus under the following accession numbers: GSE147141 (ChIP-seq) and GSE147140 (ATAC-seq).

## Supplementary Material

gkaa454_Supplemental_File
