# Supplementary material for: Regulatory sharing between estrogen receptor α bound enhancers
Source: Nucleic Acids Res. 2020 Jun 1;48(12):6597–610. doi: 10.1093/nar/gkaa454 (PMC7337896; doi:10.1093/nar/gkaa454)
Supplement: gkaa454_Supplemental_File [file gkaa454_supplemental_file.pdf]

Supplemental Material for "**Regulatory Sharing Between Estrogen Receptor α Bound Enhancers**"

Julia B. Carleton, Matthew Ginley-Hidinger, Kristofer C. Berrett, Ryan M. Layer, Aaron R. Quinlan, and Jason Gertz

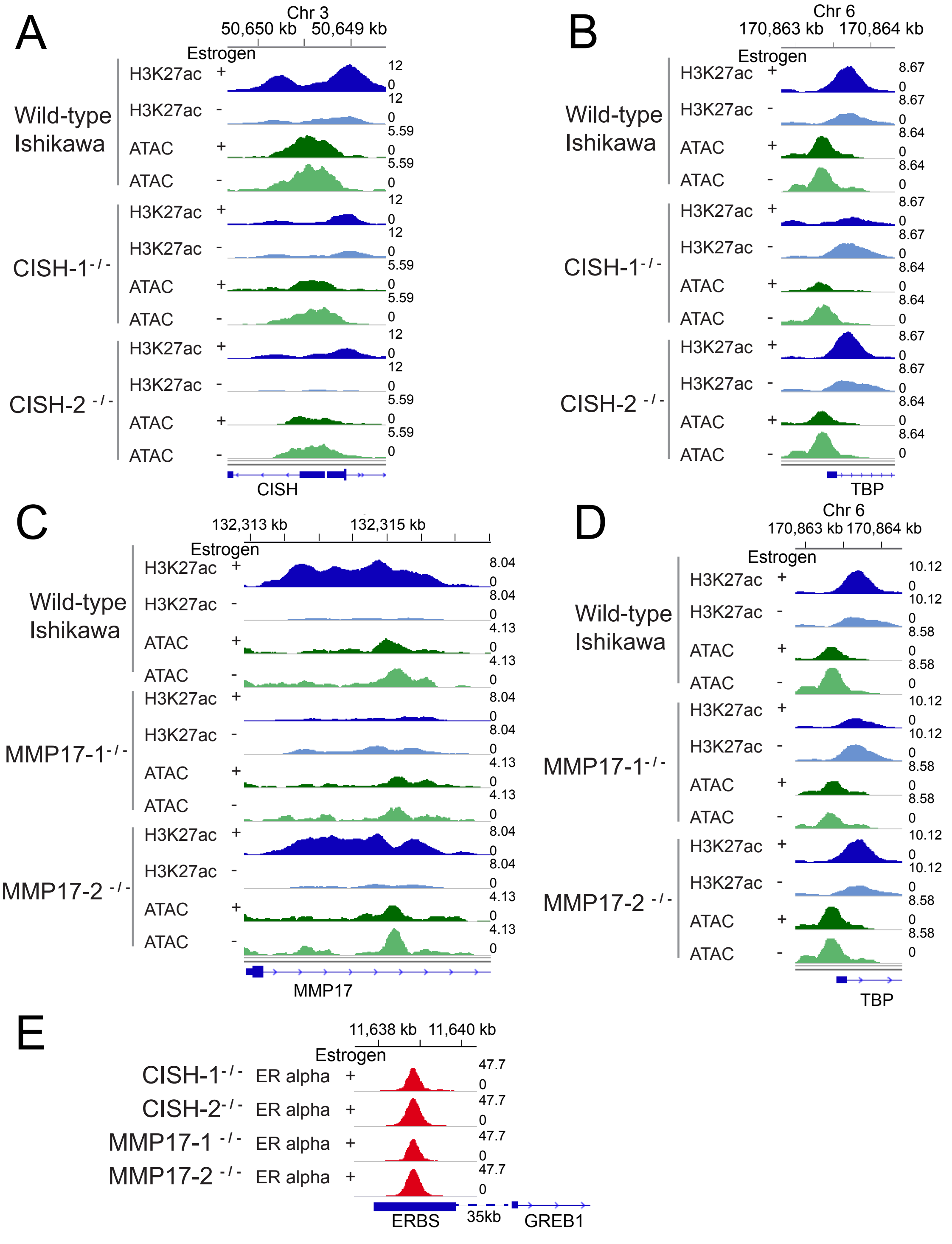

Supplemental Figure S1. ChIP-seq signal at CISH and MMP17 promoters and control regions. Genome browser tracks show H3K27ac ChIP-seq and ATAC-seq signal in the presence and absence of estrogens for the *CISH* (A) and *MMP17* (C) promoters as well as a control region, the *TBP* promoter (B and D). E) ER ChIP-seq signal in the context of E2 treatment is shown at a control ERBS upstream of *GREB1*. For all tracks, genotype information is shown on the left and track heights in reads per million are shown on the right.

A

Motif	Logo	E-value	Odds Ratio (motif)
ESR1	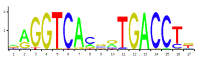	9.06e-127	4.18
Homeo domain factors	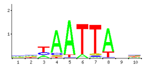	7.21e-69	1.80

B

Motif	Logo	E-value	Odds Ratio (motif)	p-value (ChIP)	Odds Ratio (ChIP)
SP1/KLF5/EGR1	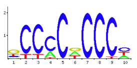	7.51e-73	1.64	1.33e-70 (EGR1)	12.79 (EGR1)
NFI	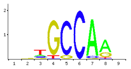	1.45e-18	1.17	5e-324 (NFIC)	6.36 (NFIC)
E-Box	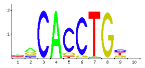	5.0e-14	1.26	5e-324 (MAX)<br>5e-324 (TCF12)<br>1.62e-72 (USF1)	14.65 (MAX)<br>11.08 (TCF12)<br>5.79 (USF1)
ETS	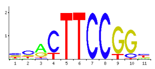	6.18e-14	1.58	5e-324 (ETV4)<br>2.8e-46 (SRF)	11.897 (ETV4)<br>8.319 (SRF)

Supplemental Figure S2. Analysis of transcription factors found at ERBS with histone acetylation. Tables show the top differentially represented motifs comparing ERBS without H3K27ac in the absence of estrogens (enriched motifs in A) and ERBS with H3K27ac in the absence of estrogens (enriched motifs in B). In panel B, the last two columns show Fisher's exact test p-values and odds ratios for the association between ChIP-seq peaks for the factor named in parentheses and H3K27ac at ERBS. Motifs were identified using AME (http://meme-suite.org/tools/ame).

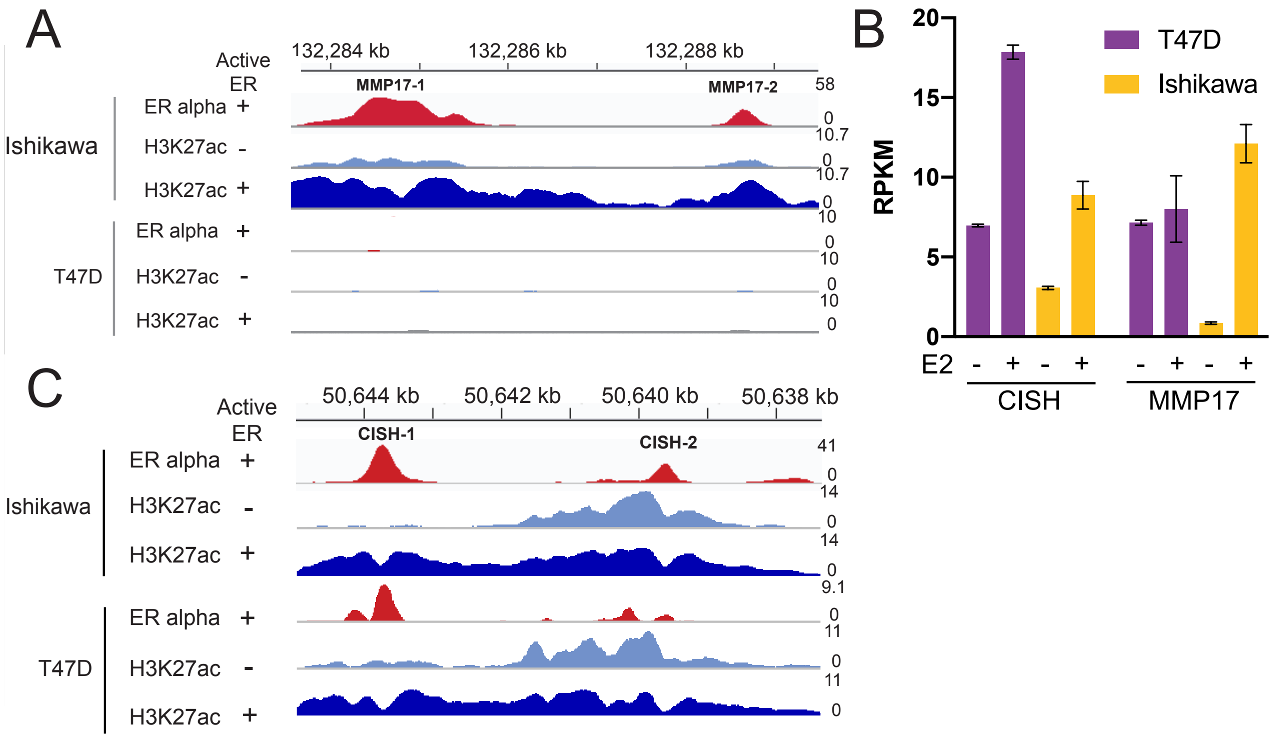

Supplemental Figure S3. Cell type-specific regulation of *MMP17* and *CISH*. Genome browser tracks show ChIP-seq signal of ER and H3K27ac in the presence and absence of estrogens for ERBS near MMP17 (A) and CISH (C) in Ishikawa and T47D, a breast cancer cell line. Track heights in reads per million are shown on the right. B) RNA-seq based gene expression levels are shown for *CISH* and *MMP17* in T47D (purple) and Ishikawa (yellow) before and after an 8-hour E2 treatment.

Table S1. Primers used

Name	Sequence (*indicates phosphothiorate modification)
qPCR_CISH_F	TGCCAGAAGGCACGTTCTTAG
qPCR_CISH_R	GCCACGAGTGGTTTTCACTG
MMP17_qPCR_F	CACTCATGTACTACGCCCTCA
MMP17_qPCR_R	TGGAGAAGTCGATCTGGATGTC
CTCF_qPCR_F	ACCTGTTCCTGTGACTGTACC
CTCF_qPCR_R	ATGGGTTCACTTTCCGCAAGG
MMP17-1_Illumina_F	ACACTCTTTCCCTACACGACGCTCTTCCGATCTGGACT CCACTGCAGGCTTC
MMP17-1_Illumina_R	GTGACTGGAGTTCAGACGTGTGCTCTTCCGATCTGCG CCATAAGGAGGAAAAAC
MMP17-2_Illumina_F	ACACTCTTTCCCTACACGACGCTCTTCCGATCTGATTG TCTCCTTCACCTGTGTT
MMP17-2_Illumina_R	GTGACTGGAGTTCAGACGTGTGCTCTTCCGATCTCGTT CCCAAGCCCTTTCC
CISH-2_Illumina_F	ACACTCTTTCCCTACACGACGCTCTTCCGATCTCCCTG GTAAGTGGTCAAGGA
CISH-2_Illumina_R	GTGACTGGAGTTCAGACGTGTGCTCTTCCGATCTAGG TGTTTTCGCCAGAATTG
CISH-1_Illumina_F	ACACTCTTTCCCTACACGACGCTCTTCCGATCTACCAG GTGAGGAACAGCTTG
CISH-1_Illumina_R	GTGACTGGAGTTCAGACGTGTGCTCTTCCGATCTTGTC TTTCCTGGATGTGGTG
KI_MMP17-1_F	G*A*G*TTGGGGCTCGTAAGGTCC
KI_MMP17-1_R	C*T*C*GGCACAGCAGGCGTTTAA
KI_MMP17-2_F	G*C*T*TGGATCACTGCCCTGTGTG
KI_MMP17-2_R	G*C*T*GGTGGAGCGACCTGG
KI_CISH-1_F	C*C*C*ATCCGCTAGGTGACTCAAAT
KI_CISH-1_R	C*T*G*TTACTGGCGGCGGC
KI_CISH-2_F	C*C*C*GCCACTTCTGAGCTGG
KI_CISH-2_R	C*A*G*AGGAGGCCGCAGGC

Table S2. Guide RNAs used

Name	Sequence with PAM	Sequence without PAM
CISH-1_396	GTCACCTAGCGGATGGGTAGTGG	TCACCTAGCGGATGGGTAG
CISH-1_586	GCCGCCGCCAGTAACAGGGAAGG	CCGCCGCCAGTAACAGGGA
CISH-2_468	AGCTCAGAAGTGGCGGGTGGAGG	GCTCAGAAGTGGCGGGTGG
CISH-2_614	CGCCGCTTCCAAGAACTCAGAGG	GCCGCTTCCAAGAACTCAG
MMP17-1_154	AGGGTCCGGCCACCTGGAGTTGG	GGGTCCGGCCACCTGGAGT
MMP17-1_300	CTGACAATGGAACTTGACCTCGG	TGACAATGGAACTTGACCT
MMP17-2_293	AGGTCGCTCCACCAGCTTCACGG	GGTCGCTCCACCAGCTTCA
MMP17-2_189	ATGTTTCCTGGTGCGTGGCTTGG	TGTTTCCTGGTGCGTGGCT
